# Searching for a Potential Blue Zone in the Nordics: A Study on Differences in Lifestyle and Health in Regions Varying in Longevity in Western Finland

**DOI:** 10.1155/jare/5535904

**Published:** 2025-08-03

**Authors:** Sarah Åkerman, Dorly Deeg, Erika Boman, Johan Niklasson, Yngve Gustafson, Fredrica Nyqvist

**Affiliations:** ^1^Social Policy, Åbo Akademi University, Vaasa, Finland; ^2^Department of Epidemiology and Data Science, Amsterdam University Medical Center, Amsterdam, the Netherlands; ^3^Department of Nursing, Umeå University, Umeå, Sweden; ^4^Department of Research and Development, Åland University of Applied Sciences, Mariehamn, Åland, Finland; ^5^Department of Community Medicine and Rehabilitation, Geriatric Medicine, Sunderby Research Unit, Umeå University, Umeå, Sweden; ^6^Department of Community Medicine and Rehabilitation, Umeå University, Umeå, Sweden

**Keywords:** ethnolinguistic, Finland, health, lifestyle, longevity, Nordic

## Abstract

To delay social and healthcare utilisation among the ageing population, there is an increasing focus on the role of health-promoting lifestyle adopted at an individual and/or community level. Longevity is generally viewed as the ultimate outcome of health, although a high life expectancy does not necessarily go together with health and/or a health-promoting lifestyle. The potential coherence between longevity, health and lifestyle may vary in different cultural, political, social and economic contexts. This Nordic regional study situated in regions differing in longevity aims to (i) explore differences in adherence to the comprehensive, health-promoting Blue Zone lifestyle principles in four regions in Western Finland (bilingual Ostrobothnia, Swedish-speaking Åland and Finnish-speaking South Ostrobothnia) and (ii) investigate regional differences in health. Thus, the present study aims to examine if adherence to Blue Zone lifestyle principles and good health is highest in the most longevous region. Survey data from the Gerontological Regional Database in 2021–2022 were used. Marginal means were calculated using ANOVA. The results showed that Åland, Finland's most longevous region, showed the best health and higher environmental agreeableness, while deviated from several Blue Zone lifestyle principles. Swedish-speaking Ostrobothnia showed good health and adherence to the Blue Zone lifestyle. South Ostrobothnia showed the poorest health but as much adherence to the Blue Zone lifestyle as Swedish-speaking Ostrobothnia. Finnish-speaking Ostrobothnia deviated the most from the Blue Zone lifestyle. The findings imply that Nordic longevous regions do not necessarily adhere to the Blue Zone lifestyle. Future research incorporating individual, community and societal factors could further elucidate whether and how longevity, lifestyle and health are interconnected in different ethnolinguistic contexts to further advance the understanding of healthy ageing and improve the implementation of effective health-promoting initiatives.

**Summary**

• Lifestyle principles important for longevity might vary in different regions.
• Åland showed the highest longevity and best health but deviated from Blue Zone lifestyle principles with the exception of environmental agreeableness.
• Swedish-speaking Ostrobothnia showed preliminary tendencies to be a longevous Blue Zone in terms of high levels of longevity, health and adherence to the Blue Zone lifestyle principles.
• Future research aiming to identify a longevous Blue Zone in Western Finland could further differentiate between the Swedish-speaking and Finnish-speaking population in Ostrobothnia in analyses of longevity.

## 1. Introduction

Due to pressures on social and healthcare systems around the world, focus is placed on healthy ageing in communities and the role of lifestyle for health promotion [1]. Longevity is often seen as the ultimate outcome of health, although in the context of highly developed social and healthcare systems in the Western world, longevity does not necessarily equate to good health and/or a health-promoting lifestyle. Nonetheless, in the 21st century, longevity is receiving increasing attention in research, policies, market and the general public—of which the concept of Blue Zones is an illustrative example [2]. Longevous Blue Zones are regions in the world characterised by extreme longevity. The originally purely demographic concept of Blue Zones [3] was launched by Poulain and colleagues who identified the first longevous Blue Zone in Ogliastra, Italy. The identification was the result of a rigorous validation process involving going through municipal birth and death registers. At present, longevous Blue Zones have been identified in Nicoya, Costa Rica; Ogliastra, Italy; Ikaria, Greece; Okinawa, Japan, and in Martinique [2]. Blue Zones can be defined as ‘a rather limited and homogenous geographical area where the population shares the same lifestyle and environment, and its longevity has been proved to be exceptionally high' [3].

While the concept of Blue Zones was originally demographic, it has expanded to include also environmental characteristics and lifestyle traits shared by the inhabitants in the longevous Blue Zones [2]. Common lifestyle traits in the Blue Zones include, amongst others, natural movement in everyday life, purpose in life, eating wisely and avoiding stress [2]. Moreover, Blue Zones are characterised by a positive attitude towards older adults with frequent interactions between the older inhabitants and the rest of the community [4]. In this sense, the Blue Zone lifestyle links with other concepts conducive to healthy ageing such as age-friendly cities [5] and active ageing [6]. The Blue Zone lifestyle also shares several similarities with the Stanford Lifestyle Medicine Pillars in terms of, for example, physical lifestyle, healthful nutrition and social connection [2]. Both frameworks aim at behavioural change, with the former aiming at public health on the community level while the Stanford Lifestyle Medicine Pillars focus on individual care [2]. The Blue Zone lifestyle principles also share both similarities and differ from the Power9-framework suggested by Buettner & Skemp [7], as the Power9 includes elements that have not been scientifically observed in longevous Blue Zones according to Poulain & Herm [2].

Despite the growing interest of health-promoting lifestyle frameworks, the evidence supporting the idea that specific lifestyle patterns can universally explain regional variations in longevity remains limited [8, 9]. More research is therefore needed to explore how combinations of health behaviours, social environments and demographic factors contribute to longevity in various regions that differ socially, culturally, politically and economically. The aim of the present study is therefore to, inspired by the Blue Zone framework, explore whether and how longevity, health and lifestyle go together in a regional comparison in a Nordic setting. A region demonstrating coherence across these three domains could offer preliminary insights into the conditions that may support healthy ageing at a population level. Additionally, incorporating longevity, health and lifestyle also offers a more nuanced understanding of healthy ageing and may provide new insights to the potential of lifestyle-promoting initiatives [1, 2]. The Blue Zone lifestyle framework further captures behavioural, cognitive, emotional and social aspects of well-being at both individual and community levels—broadening the scope of what constitutes healthy ageing beyond individual biomedical indicators alone. Thus far, no studies have investigated the applicability of the Blue Zone framework for longevous regions in the Nordics.

This regional study brings forward the case of Finland that differs from the previously identified longevous Blue Zones in terms of, for example, a social democratic welfare regime [10] and a sub-arctic climate. Finland gained independence in 1917 after breaking free from Russia. The country's history of multiple wars, including a civil war during the 20th century, in combination with the partly arctic climate is often used as a backdrop for presenting the stereotypical introverted Finnish mentality especially attributed to men [11]. This cultural narrative is sometimes linked to Finland's relatively high levels of suicide in a European Union comparison [12]. In contrast, Finland has been ranked as the happiest country in the world several times, potentially due to its stable governance, high levels of equality and educational attainment and little corruption [13]. Additionally, Finland possesses health advantages such as lower prevalence of smoking and diabetes and a slightly higher life expectancy than the European Union average [12]. Despite its universal social and healthcare system grounded in a strong commitment to equality throughout the life course, there are considerable regional differences in longevity and health in Finland [14] as well as in other Nordic countries such as Denmark and Sweden [15].

This study explores bilingual Western Finland, considering that Swedish speakers in Western Finland, particularly men, have a higher longevity compared to Finnish speakers living in the same region [16–18]. The Swedish speakers' health advantages are believed to be the result of both social and heredity factors [16–18]. Previous studies on ethnolinguistic differences in lifestyle in the bilingual region of Ostrobothnia in Western Finland have shown that older Swedish speakers are more civically engaged and possess higher levels of social capital than their Finnish-speaking peers which could tentatively contribute to, or reflect, better health among older Swedish speakers [19]. The present study compares unilingual Swedish-speaking Åland, bilingual Ostrobothnia and unilingual Finnish-speaking South Ostrobothnia. The Åland Islands were ranked as one of the most longevous NUTS regions in Europe in 2021 with a life expectancy of 84.6 and in 2023 with 85.1 [20]. Unilingual Finnish-speaking South Ostrobothnia represents an average healthy region in Finland, while bilingual Ostrobothnia has been noted for its overall healthier population [14]. Regional comparisons of longevity1 in Finland during recent years suggest that Åland is the most longevous area, closely followed by Ostrobothnia (Table 1). South Ostrobothnia is closer to the Finnish national average longevity (Table 1). The present study is the first to use the Blue Zone framework to explore how regions that differ in longevity correlate with regional differences in comprehensive, health-promoting lifestyle and health in Western Finland.

## 2. Aim and Research Questions

The aim of this study is to explore regional differences in adherence to the Blue Zone lifestyle principles (inspired by the seven Blue Zone principles suggested by Poulain & Herm [2, 3]) and selected health indicators in various longevous areas in Western Finland. The Blue Zone principles assessed in this study include Move naturally, Eat wisely, Avoid stress and get plenty of sleep, Strong family ties and community support, Respect the planet, A purpose in life and a supplementary principle of environmental features. In addition, based on general studies on longevity, we investigate health indicators such as instrumental activities of daily living (IADL), personal activities of daily living (PADL), dental health, self-rated health, pain, medical conditions, number of medicines, fatigue and cognitive health.

The research questions are as follows:

- Are regions with higher longevity in Western Finland characterised by greater adherence to the Blue Zone principles?
- Are regions displaying higher longevity in Western Finland characterised by better health?
- Is there a region in Western Finland that shows preliminary tendencies to be a longevous Blue Zone in terms of high levels of longevity, health-promoting lifestyle adherence and health?

## 3. Data and Methods

**Figure 1:**
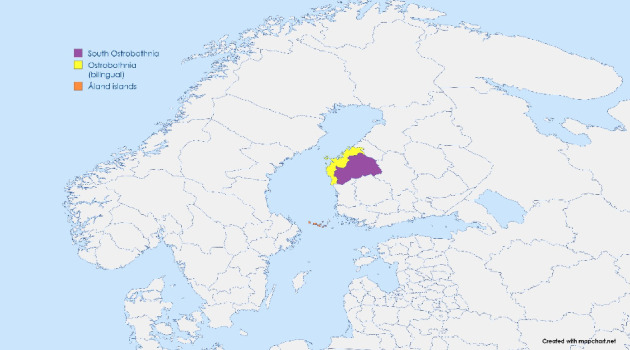
A map of the studied geographical regions in Western Finland.

Regional adherence to the Blue Zone principles and selected health indicators was investigated by using data from the multidisciplinary Gerontological Regional Database2 (GERDA) survey in 2021/2022 [21]. In the wave collected in 2021/2022, the GERDA questionnaire was sent out to older adults in the regions of Åland, Ostrobothnia and South Ostrobothnia in Finland and in Northern Sweden. For the present study, only the data collected in Finland were used. A geographical overview of the regions is provided in Figure 1. In the GERDA wave in 2021/2022, the questionnaire was sent out by post to every individual born in 1930, 1935, 1940, 1945, 1950 and 1955, except for the city of Vaasa, Ostrobothnia and in South Ostrobothnia, where every second individual was selected. In Åland, the questionnaire was sent to 1349 individuals and 831 responded (response rate 61.6%). In Ostrobothnia, the questionnaire was sent to 7272 individuals and 3363 responded (response rate 46.2%), and in South Ostrobothnia to 5981 individuals and 2732 responded (response rate 45.7%).

When identifying Blue Zones, the regions were counted as three (Åland, Ostrobothnia and South Ostrobothnia) due to lack of separate statistical data on longevity among Swedish and Finnish speakers. When investigating adherence to the Blue Zone principles and selected health indicators using GERDA data, the geographic region of Ostrobothnia was treated as two separate regions based on language group affiliation. Therefore, analyses based on the GERDA survey data include four regions (Åland, Swedish-speaking Ostrobothnia, Finnish-speaking Ostrobothnia and South Ostrobothnia) in the present study. Similar divisions of the regions have been used for analyses in previous studies using GERDA data, for example, in studies on loneliness [22], morale [23] and informal caregiving [24]. Additionally, while the Blue Zone lifestyle has not been the focus of previous studies using GERDA data, several social- and health-related variables used in the present study have been categorised and treated in a similar manner as, amongst others, the aforementioned studies on loneliness [22], morale [23] and informal caregiving [24]. The treatment of variables based on the GERDA survey in the present study will be further detailed in Section 3.1.

### 3.1. Indicators for Investigating Adherence to Blue Zone Principles and Selected Health Indicators in the GERDA Survey

#### 3.1.1. Covariates

Region was treated as the independent variable. To provide a comprehensive analysis, we included various control variables that could influence the outcomes. These control variables included age (66, 71, 76, 81, 86, 91), gender, educational level and personal income. Gender was dichotomised into two answering options (male, female) with ‘I don't know' (*N* = 19) treated as missing values. Educational level was categorised into three categories (low, medium and high). A low level represents 0–9 years of education, while a medium level represents 10–12 years, and a high level refers to more than 12 years. Personal income was categorised into three levels (0–1000 euros, 1001–2000 euros and more than 2000 euros).

#### 3.1.2. Blue Zone Lifestyle Principles

The number of variables contributing to a Blue Zone lifestyle principle ranged from 1 (*Eat wisely*) to 10 (*Strong family ties and community support*).

##### 3.1.2.1. Move Naturally

Being physically active was assessed as performing moderately demanding activity for 150 min or more a week and/or as performing 75 min or more of very demanding physical activity, in line with recommendations by the World Health Organisation [25]. This was assessed by creating a sum variable (frequency multiplied by minutes) out of the following four questions: ‘How often have you carried out any demanding physical activity such as fast walking, raking leaves, washing windows, bicycling, swimming or other exercise at moderate pace during the last seven days?' (0, 1, 2, 3, 4, 5, 6, 7, more than 7 times), ‘How long did the moderately demanding activity take place on average?' (0, 10, 20, 30, 40, 50, 60, more than 60 min), ‘How often have you carried out any very demanding physical activity such as heavy construction or gardening work, chopping wood, intense Nordic walking, running or other exercise at high pace during the last seven days?' (0, 1, 2, 3, 4, 5, 6, 7, more than 7 times) and ‘How long did the very demanding activity take place on average?' (0, 10, 20, 30, 40, 50, 60, more than 60 min).

The question on household animals was extracted from the Modified Norling Petterson Selander (MNPS) Interest Checklist [26]. Performance was assessed independently for each item with the question ‘Do you perform this activity?' (yes, no).

##### 3.1.2.2. Eat Wisely

Eating wisely was assessed with the body mass index (BMI) and reporting underweight, healthy and overweight. BMI was calculated by using the formula: weight (kg)/(height ∗ height) (m). When calculating height and weight, those reporting height below 100 or above 210 cm (*N* = 1) or those weighing less than 35 kg or more than 210 kg (*N* = 3) were excluded. Following the Finnish recommendations for interpreting BMI scores for older adults [27], the variable was used as a categorical variable (‘underweight' [10–22.9), ‘healthy weight' [23–29.9] and ‘overweight' [30–70]).

##### 3.1.2.3. Avoid Stress and Get Plenty of Sleep

Sleep quality was assessed with the question ‘Do you have good night sleep?' which was dichotomised into ‘no' and ‘yes', while ‘I don't know' (*N* = 522) was treated as missing. In a similar manner as in the study by Hörnsten, Lövheim, Nordström et al. [28], depression was assessed by a combined variable consisting of the one-item question ‘Are you depressed/gloomy?' (no, yes) and the Geriatric Depression Scale (GDS) 4-item version [29] comprising of four yes/no questions: ‘Are you basically satisfied with your life?', ‘Do you feel that life is empty?', ‘Are you afraid that something bad is going to happen?' and ‘Do you feel happy most of the time?'. A respondent was categorised as depressed when scoring two points or more on GDS4 and/or answered ‘yes' to the one-item question.

Happiness was assessed with the question ‘How happy or unhappy are you feeling at the moment?' (very unhappy, rather unhappy, hard to say, rather happy, very happy). The variable was treated as a 1–5 scale with 5 representing very happy. Financial strain was measured by the question ‘Is it possible for you to make ends meet?', with four answering options. The variable was dichotomised into ‘without difficulty' and ‘with difficulty' (with some difficulty, with difficulty, with much difficulty).

##### 3.1.2.4. Strong Family Ties and Community Support

Civil status was dichotomised into two categories (‘widowed, single, unmarried' vs. ‘married, cohabiting partner, outside-of-household partner'). Cohabiting with someone was assessed with the multi-response question ‘Do you live with someone?' (yes, with spouse or partner/sibling/child/grandchild/other relative/someone vs. no, I live alone). The question was dichotomised into ‘lives alone' and ‘cohabits with someone'.

Two variables were used to measure contact frequency with family members and relatives (child, grandchild, sibling, parent, other relative) and with neighbours and friends, respectively. The question ‘How often are you in contact with one/several of the following persons?' had five answering options. ‘Infrequent contact' (several times a month, a few times a year, never, the person does not exist) indicated contact with someone in the category less often than several times a week. ‘Frequent contact' (several times a week) indicated that the respondent had contact with at least one person in the category several times a week.

Number of confidants was assessed with the question, ‘Do you have a confidant with whom you can speak about anything that is sharing both concerns and joys?' The answer alternatives included multiple options of relatives, friends, neighbours, staff and unspecified confidants. After calculating the median score, the variable was dichotomised into ‘0–1 confidants' and ‘2 confidants or more'.

Volunteering was assessed with the question ‘Do you engage in any kind of voluntary, unpaid work for any association? (for example, social organisation or aid organisation, religious organisation, sports organisation, cultural association)' (no, yes).

Loneliness was measured with the question ‘Do you suffer from loneliness?' (yes, no).

A positive attitude towards older people in the neighbourhood was assessed by an item extracted from a nonstandardised scale assessing ageism in various settings. The question was ‘What kind of attitude towards older people do you think that there is in society?' (I don't know, negative, neutral, positive). The variable was dichotomised into ‘positive' versus ‘other' (I don't know, negative, neutral). The variable ‘Most people in this area can be trusted' was dichotomised into ‘other' (strongly disagree, disagree, agree) and ‘strongly agree' (strongly agree) after checking their distribution.

##### 3.1.2.5. Respect the Planet

Forest work and gardening were questions extracted from the MNPS Interest Checklist [26] described in Move naturally.

##### 3.1.2.6. A Purpose in Life

Sense of purpose was assessed with the question ‘Do you feel needed?' (no, yes) and with the question ‘How meaningful do you experience your life at the moment?' (very meaningless, rather meaningless, hard to say, rather meaningful, very meaningful). Meaningfulness was assessed as a continuous variable with possible scores of 1–5, with a higher score representing more meaningfulness. Morale was assessed using the Philadelphia Geriatric Center Morale Scale (PGCMS) [30]. The instrument consists of 17 questions with the answer alternatives ‘yes' and ‘no'. The scale has a maximum of 17 points, where each answer indicating high morale gives one point. The instrument was treated as a scale. Religious activity was extracted from the MNPS Interest Checklist [26] and treated as described in the ‘1. Move naturally' principle. Belief in a higher power was assessed with the question ‘Do you believe in God or a higher power' (no, yes).

#### 3.1.3. Additional Principle: Environmental Features

Variables assessing perceived environmental facilitators for outdoor mobility were selected from the original 16-item instrument Perceived Environmental Facilitators (PENFOM) [31]. The question was ‘Are there factors in your living place and in the nearby area that entice to being physically active outdoors? (for example, closeness to green spaces, the condition of the streets)' (no, yes). The variable ‘I really feel part of this area' was dichotomised into ‘other' (strongly disagree, disagree, agree) and ‘strongly agree' (strongly agree) after checking the distribution.

#### 3.1.4. Health Variables

IADL were assessed with the following questions: ‘Do you clean your dwelling (vacuum and wipe the floor) without help from others?' ‘Do you do grocery shopping without help from others?' ‘Do you use public transportation such as buses, planes or trains without help from others?' and ‘Do you cook without help from others?' (yes/no). A sum variable with a scale of 0–4 was created, with a higher score suggesting more dependency. PADL were assessed with the question ‘Do you shower without help from another person?' (yes, no) with ‘no' indicating dependency.

Medical conditions were assessed using questions on whether the respondent had had a stroke, myocardial infarction or whether the respondent was currently experiencing joint issues, diabetes, high blood pressure and/or using blood pressure medication. The sum variable was treated as a scale (0–5 points) with a higher score indicating more medical conditions.

Number of medications was assessed with the question ‘How many different medicines from the pharmacy do you regularly take?' and was treated as a continuous variable with possible scores ranging from 0 to 30. Counts above 30 were excluded from the analysis (*N* = 1). Dental health was assessed with the questions ‘Have you visited a dentist during the last 12 months?' (no/yes) and ‘Do you mostly have your own teeth?' (no, yes) with ‘yes' indicating dental health. Self-rated health was assessed with the following question: ‘In general, how would you say your health is?' (poor, fair, good, very good, excellent) and was treated as a scale with higher scores suggesting better health. Pain was assessed with the following question 'Have you had pain/ache during the last week?' (yes, no). Good memory was assessed with the question ‘Do you experience that you have a bad memory?' and was dichotomised into ‘poor memory' (yes, and it affects daily life; yes, but it does not affect daily life) and ‘good memory' (no). Fatigue was assessed with the question ‘Do you feel tired and lacking strength?' (yes, no).

### 3.2. Analyses

Longevity was explored by using official statistics and is presented in Table 1. To explore statistical differences in the distribution of background variables among the four regions using GERDA data, estimated marginal means (EMMs), confidence intervals and *F*-values were derived from ANOVA (Table 2).

To compare adherence to the Blue Zone principles and selected health indicators, analyses were performed by using data from the GERDA survey (Table 3). Region was treated as the independent variable. EMMs, confidence intervals and *F*-values were derived from ANOVA. The analyses controlled for age, gender, educational level and personal income. In case of significant regional differences, the region adhering to the Blue Zones principles the most contributed to the Blue Zone score. Each Blue Zone principle was awarded one point in total so that the contribution of each item was calculated by dividing 1 by the number of included items within each Blue Zone principle. The same procedure was conducted for health indicators, with each health indicator calculated as 1 point.

All analyses were conducted in Excel and SPSS 29 (IBM Corp. Released 2023. IBM SPSS Statistics for Windows, Version 29.0.2.0, Armonk, NY: IBM Corp).

## 4. Results

When analysing background variables in the GERDA sample (Table 2), the results from the ANOVA showed that Åland (47% men) and Swedish-speaking Ostrobothnia (46% men) had a slightly more even distribution of female and male respondents than Finnish-speaking Ostrobothnia (41% men) and South Ostrobothnia (43% men). On average, the sample on Åland was statistically significantly younger (EMM 2.50, 95% CI 2.40–1.59), had a higher educational level (EMM 1.98, 95% CI 1.94–2.02) and a higher monthly personal income (EMM 2.23, 95% CI 2.20–2.27) as compared to the other regions.

Table 3 shows adherence to the Blue Zone principles and health indicators in the four regions based on GERDA. Swedish-speaking Ostrobothnia and South Ostrobothnia clearly adhered to the Blue Zone principles the most with a total Blue Zone score of 1.73, while Finnish-speaking Ostrobothnia (with a Blue Zone score of 0) deviated the most. Åland scored 1.23. Notably, Swedish-speaking Ostrobothnia showed high scores on the Blue Zone principles of Avoid stress and get plenty of sleep, Strong family ties and community support, and A purpose in life. South Ostrobothnia also adhered to the Blue Zone principles of Strong family ties and community support and A purpose in life—and additionally also Respect the planet. Åland scored high on environmental features. Finnish-speaking Ostrobothnia did not score a single point on adherence to the Blue Zone lifestyle principles. Åland showed the best health, followed by Swedish- and Finnish-speaking Ostrobothnia. South Ostrobothnia showed the poorest health.

## 5. Discussion

The main aim of the present study was to explore regional differences in comprehensive, health-promoting lifestyle and health in four regions in Western Finland that differ in longevity and to explore whether they cluster in regions with greater longevity—thus providing preliminary clues for a potential Blue Zone. Thus far, the Blue Zone principles and their potential correlation with regional differences in longevity and health have not been investigated in a Nordic context. When comparing adherence to health-promoting lifestyle factors and selected health indicators, the results revealed a mixed pattern. Swedish-speaking Ostrobothnia and South Ostrobothnia demonstrated the highest adherence to lifestyle principles associated with longevity, while Åland showed the most favourable health outcomes. Swedish-speaking Ostrobothnia might be deemed a potential longevous Blue Zone in terms of being located in a longevous area and scoring comparatively high on both health-promoting lifestyle and health. However, future rigorous demographic research is needed in order to validate exceptional longevity in this region, and especially in the Swedish-speaking population in Ostrobothnia. Åland was the most longevous and showed the best health, but deviated from several Blue Zone lifestyle principles with the exception of environmental agreeableness.

In line with previous research, older adults in Swedish-speaking Ostrobothnia were more inclined to volunteer and be socially active [19], and less likely to report depression and loneliness than their Finnish-speaking peers [22]. It is possible that the ethnolinguistic context of bilingual Ostrobothnia boosts social interactions and civic engagement among the Swedish speakers. Being part of an ethnolinguistic minority could, on the one hand, constitute barriers to receiving services in one's mother tongue, but on the other hand, also foster social cohesion and social capital [19]. Volunteering and keeping in contact with family members could be seen as paralleling the high levels of subjective well-being in Swedish-speaking Ostrobothnia, including eudaimonic dimensions referring to cognitive aspects of well-being involving meaningfulness and reaching potential. Eudaimonic well-being has been investigated scarcely in the Blue Zone literature, although one Italian study found that eudaimonic well-being was higher in the Italian Blue Zone area in comparison to non-Blue Zones [32]. High levels of morale, which are considered a measure of well-being, have been found to increase the probability of survival among older adults in Sweden and Finland [33].

Swedish-speaking Ostrobothnia and South Ostrobothnia are traditionally seen as part of the Finnish ‘bible belt' with higher levels of religiousness. Åland and Finnish-speaking Ostrobothnia represented in this aspect are more secularised areas with lower levels of religiousness. Favourable health effects of religiousness have been identified in several studies; for example, in the Blue Zone in Ogliastra on Sardinia, Italy, religiousness was found to be strongly associated with life satisfaction [34]. Higher levels of religiousness were also reported in the relative Blue Zone as compared to non-Blue Zones in the Netherlands [9].

When considering the total score for the environmental features, Åland ranked highest. The positive environmental aspects may be partly attributed to its archipelago setting and relative remoteness, which tentatively boosts social cohesion among the inhabitants. On Åland, it was most common to report beautiful scenery and other positive attributes regarding the physical environment, possibly reflecting close bonds between nature and the inhabitants on an archipelago in comparison with inhabitants living on the mainland. These findings suggest that island environment may contribute to a heightened sense of environmental well-being compared to mainland regions. Notably, several of the identified longevous Blue Zones around the world are located on islands or peninsulas where geographic isolation and close-knit communities may play a role in promoting health and longevity [2, 3]. Similarly, environmental agreeableness was also reported in the relative Blue Zone discovered in the Netherlands [9], further supporting the potential relevance of environmental context in shaping healthy ageing outcomes.

Regarding health, South Ostrobothnia ranked lowest with the highest prevalence of pain and poor dental health, the highest number of medical conditions and medicine use and the lowest prevalence of good memory. The largest regional differences in health were the ones assessing dental health, which is an important finding given the links between dental health and survival [35]. In South Ostrobothnia, low scores on health indicators could be seen in line with the region also displaying the lowest longevity. The region with the highest longevity also showed the best health—Åland showed the best dental health, least pain, few medical conditions, low number of medications and better memory. In contrast, Åland showed lower scores on self-rated health than in the other regions. Åland has been found to differ from the other regions not only genetically but also culturally [16, 36], and it is possible that the unique characteristics of Åland may play a significant role in shaping how residents perceive their health status. Indeed, correlates of self-rated health also vary depending on context [37].

Summarising, there were no clear patterns in terms of regional differences in coherence of longevity, health-promoting lifestyle and health. While one might expect regions with higher longevity to also exhibit stronger alignment with health-promoting lifestyle principles, this was not uniformly the case. Swedish-speaking Ostrobothnia (and South Ostrobothnia) demonstrated the highest adherence to such lifestyle and ranked second in terms of health indicators. South Ostrobothnia, in contrast, showed the poorest health and is a less longevous area than the other studied regions. Finnish-speaking Ostrobothnia deviated the most from the Blue Zone lifestyle, but due to limitations in official statistics, it was not possible to assess potential ethnolinguistic differences in longevity in Ostrobothnia. Åland, on the other hand, had the highest longevity and the best health but deviated from several lifestyle principles commonly associated with long-lived populations. This suggests that longevity and health on Åland may be influenced by other factors. Environmental agreeableness, another feature highlighted in the Blue Zones research by Poulain et al. [3], although not explicitly included in the seven Blue Zone lifestyle principles [2], was particularly distinct for Åland, potentially offering an alternative explanation for its favourable ageing profile.

Blue Zones have been criticised for overlooking the economic and political context that shapes health and longevity [8, 38]^,^. Differences in health and longevity on Åland could be attributed to the higher availability of social and healthcare compared to the other regions [14]. The descriptive characteristics showed that respondents on Åland had higher levels of education and personal income than in the other regions. Socioeconomic status has been found to influence mortality in other Nordic equality-striving welfare states as well [15]. Other possible explanations for longevity in Blue Zones include high rates of inbreeding in combination with low immigration rates that might have resulted in a favourable genetic pool [2]. There are documented genetic differences between Swedish-speaking and Finnish-speaking Finns and between inhabitants on Åland and mainland Finland [16, 17], but the implications for longevity and health have been scarcely investigated thus far. This could be a topic for future research. In the case of immigration per se, Åland stands out with a lower prevalence of individuals born in the area according to national statistics. High longevity on Åland could in this case also be explained by a healthy and resourceful pool of immigrants. Further research is needed to fully understand the role of both genetic and socioeconomic resources for longevity on Åland.

Ethnolinguistic differences in life expectancy were not investigated in the present study, but the analyses of adherence to the Blue Zone principles showed clear differences among older adults in Swedish-speaking versus Finnish-speaking Ostrobothnia. The prevalence of people born in the area was the lowest in Finnish-speaking Ostrobothnia. Future research on differences in health and longevity in bilingual environments could further delve into the role of migration or lack of migration, for adapting a certain lifestyle in old age. Additionally, to acquire a comprehensive, contextual understanding of regional differences in longevity, health and/or lifestyle, future research could further adopt a socioecological framework [39] including markers on gender inequality, internet literacy and environmental health. Such a framework could also arguably be useful for further contextualising longevity in the longevous Blue Zones around the world.

### 5.1. Strengths and Limitations

The GERDA survey enabled dividing Ostrobothnia into two regions based on linguistic affiliation. The multidisciplinary GERDA survey further enabled the application of an analytical framework inspired by the Blue Zone principles, allowing the investigation of proxies for nearly all lifestyle-related factors—except for Ensure the legacy for future generations. GERDA is based on a self-response procedure which may cause sample bias as healthier adults are more likely to participate than frail older adults. The risk of such bias can be deemed even higher in the Finnish-speaking regions where the response rates were lower and the sample thus tentatively even more selective. However, Finnish-speaking regions did not show better levels of health. Another limitation with GERDA is that the data collection was in Winter/Spring in 2021/2022 during the third wave of the COVID-19 pandemic. Social restrictions, mostly applied locally depending on incidence rates during the third wave of the pandemic, may have affected the responses in unknown ways.

In the present study, using official statistics, the region of Ostrobothnia was not distinguished into Swedish- and Finnish-speaking regions. It remains to be investigated whether life expectancy in this region was higher in the Swedish-speaking part than in the Finnish-speaking part of the population. Nonetheless, by using publicly available population-level data, it is not feasible to trace individual origin and migration events. A more rigorous demographic procedure, including the Extreme Longevity Index [2, 3], would be needed to determine whether an actual longevous Blue Zone could be identified in Western Finland.

## 6. Conclusions

This Nordic regional study observed associations between longevity and health, while lifestyle-related factors were more divergent. Åland was the most longevous region and showed the best health in Western Finland, but did not uniformly align with the Blue Zone lifestyle principles. Tentatively, in Western Finland, high longevity could go together with adherence to a religious, meaningful and socially active lifestyle or alternatively with living in a physically and socially agreeable environment. Future research could further apply socioecological frameworks when exploring coherence of longevity, health-promoting lifestyle and health to provide further insights into healthy ageing in different ethnolinguistic, social, cultural, political and/or economic contexts. Additionally, future research with a more rigorous demographic procedure, including a differentiation between Swedish- and Finnish-speaking Ostrobothnia in analyses of longevity, could further determine whether there exists a longevous Blue Zone in Western Finland—possibly in Swedish-speaking Ostrobothnia.

## Figures and Tables

**Table 1:** Regional comparison of longevity in Western Finland based on official statistics.

	Åland Islands	Ostrobothnia	South Ostrobothnia		Finland
Life expectancy among newborns					
2020–2022	83.47	83.10	81.83		81.60
Ranking	1	2	3		
2017–2019	83.48	82.95	82.08		81.65
Ranking	1	2	3		
Life expectancy among newborns (female)					
2020–2022	85.84	85.21	84.49		84.28
2017–2019	85.79	85.10	84.96		84.34
Life expectancy among newborns (male)					
2020–2022	81.17	81.05	79.24		78.93
2017–2019	81.20	80.84	79.26		78.92
Life expectancy at age 65					
2018–2022	21.06	21.07	20.30	Year: 2022	19.71
Ranking	2	1	3		
2015–2019	20.72	21.07	20.32	Year: 2019	20.41
Ranking	2	1	3		
Life expectancy at age 65 (female)					
2018–2022	22.48	22.56	21.89	Year: 2022	21.32
2015–2019	22.22	22.52	22.03	Year: 2019	22.02
Life expectancy at age 65 (male)					
2018–2022	19.63	19.51	18.56	Year: 2022	17.94
2015–2019	19.16	19.48	18.40	Year: 2019	18.55
Life expectancy at age 80					
2018–2022	9.87	9.66	9.35	Year: 2022	8.91
Ranking	1	2	3		
2015–2019	9.55	9.67	9.34	Year: 2019	9.47
Ranking	2	1	3		
Life expectancy at age 80 (female)					
2018–2022	10.63	10.36	10.10	Year: 2022	9.63
2015–2019	10.39	10.39	10.07	Year: 2019	10.17
Life expectancy at age 80 (male)					
2018–2022	8.96	8.75	8.27	Year: 2022	7.90
2015–2019	8.50	8.65	8.22	Year: 2019	8.41
Rate of 90+-year-olds in 2022					
Number of individuals aged 90 and over in 2018 divided by the number of individuals aged 60–69 years in 1992	336/2186	2305/16,382	2605/21,961		58,164/481,188
Rate	0.15	0.14	0.12		0.12
Ranking	1	2	3		
Rate of 90+-year-olds in 2018					
Number of individuals aged 90 and over in 2018 divided by the number of individuals aged 60–69 years in 1988	312/2276	2239/16,679	2520/21,874		51,572/459,734
Rate	0.14	0.13	0.12		0.11
Ranking	1	2	3		
Rate of 95+-year-olds in 2022					
Number of individuals aged 95 and over in 2022 divided by the number of individuals aged 60–69 years in 1987	91/2286	536/16,575	562/21,391		11,857/451,400
Rate	0.040	0.032	0.026		0.026
Ranking	1	2	3		
Rate of 95+-year-olds in 2018					
Number of individuals aged 95 and over in 2018 divided by the number of individuals aged 60–69 years in 1983	66/2370	478/16,376	556/20,412		9923/425,019
Nonagenarian rate	0.028	0.029	0.027		0.023
Ranking	2	1	3		
Total score	**14**	**16**	**30**		
Total ranking	**1**	**2**	**3**		

*Note:* The regional comparison of longevity was calculated and ranked based on five criteria: the highest life expectancy among newborns, 65-year-olds and 80-year-olds, and the rate of 90+-year-olds and 95+-year-olds, respectively. Based on the five criteria, a total score and ranking were calculated and presented in bold text. The number of individuals aged 90+ and 95+ was divided by the number of individuals aged 60–69 before 35 years. By considering the latter age group, population instability was minimised, as migration mostly takes place at younger ages. Male and female life expectancies were reported, although only total life expectancy was ranked. The lower the score, the higher the ranking (and thus the most longevous region). A national average was included for comparison. Longevity in Swedish- versus Finnish-speaking Ostrobothnia was not possible to compare in the data used.

**Table 2:** Estimated marginal means (EMM), 95% confidence intervals (CIs) and *F*-value for regional distribution of background variables and descriptive analysis of population stability in the Gerda survey.

	Åland (*N* = 831)	Swedish-speaking Ostrobothnia (*N* = 2123)	Finnish-speaking Ostrobothnia (*N* = 1240)	South Ostrobothnia (*N* = 2732)	*F* value	*p* value
	EMM (95% CI)	EMM (95% CI)	EMM (95% CI)	EMM (95% CI)		
Sex						
Male	0.47 (0.44–0.51)	0.46 (0.44–0.48)	0.41 (1.39–0.44)	0.43 (0.41–0.45)	3.85	0.01
Age						
1–6, with 6 representing the oldest age group	2.50 (2.40–2.59)	2.70 (2.64–2.76)	2.67 (2.59–2.74)	2.60 (2.55–2.65)	4.95	0.00
Educational level						
1–3, with 3 representing the highest level of education	1.98 (1.94–2.02)	1.74 (1.71–1.76)	1.72 (1.68–1.75)	1.76 (1.73–1.78)	34.37	≤ 0.001
Personal monthly income after taxes						
1–3, with 3 representing the highest income	2.23 (2.20–2.27)	1.97 (1.95–2.00)	2.03 (1.99–2.06)	1.95 (1.93–1.97)	60.02	≤ 0.001
Population stability						
Born in the area: yes % (*N*)	32.6 (269)	51.8 (1095)	27.8 (341)	44.4 (1206)		

*Note:* Age represents 1 = 66, 2 = 71, 3 = 76, 4 = 81, 5 = 86, 6 = 91. Educational level represents 1 = low level of education [0–9 years of education], 2 = medium level [10–12 years of education], 3 = high level [more than 12 years of education]. Personal income represents 1 = 0–1000 euros, 2 = 1001–2000 euros, 3 = more than 2000 euros.

**Table 3:** Estimated marginal means (EMM), 95% confidence intervals (CIs) and *F*-value for regional adherence to Blue Zone principles and selected health indicators based on the GERDA survey.

	Åland (*N* = 831)	Swedish-speaking Ostrobothnia (*N* = 2123)	Finnish-speaking Ostrobothnia (*N* = 1240)	South Ostrobothnia (*N* = 2732)	*F*-value	*p*-value
	EMM (95% CI)	EMM (95% CI)	EMM (95% CI)	EMM (95% CI)		
1. Move naturally						
≥ 150 min of moderate and/or 75 min of intensive exercise weekly: yes	0.62 (0.58–0.66)	0.66 (0.64–0.68)	0.61 (0.59–0.64)	0.60 (0.59–0.62)	5.20	≤ 0.001
Household animals: yes	0.50 (0.43–0.57)	0.40 (0.35–0.44)	0.45 (0.39–0.50)	0.37 (0.33–0.41)	4.52	≤ 0.001
Accommodation: house	0.75 (0.72–0.78)	0.81 (0.79–0.83)	0.69 (0.67–0.71)	0.86 (0.84–0.87)	52.44	≤ 0.001
Blue Zone score	0.33	0.33	0	0.33		
2. Eat wisely						
Healthy BMI (23–29.9): yes	0.64 (0.60–0.67)	0.61 (0.59–0.63)	0.62 (0.59–0.65)	0.62 (0.60–0.64)	0.46	0.71
Blue Zone score	0	0	0	0		
3. Avoid stress and get plenty of sleep						
Sleeps well: yes	0.71 (0.68–0.75)	0.72 (0.70–0.74)	0.73 (0.70–0.76)	0.72 (0.71–0.74)	0.17	0.92
Depression: yes	0.16 (0.14–0.18)	0.12 (0.11–0.14)	0.13 (0.11–0.15)	0.14 (0.13–0.15)	2.70	0.04
Happiness (1–5)	3.89 (3.85–3.94)	3.93 (3.90–3.96)	3.93 (3.89–3.97)	3.95 (3.92–3.97)	1.40	0.24
Financial strain (1–4)	1.46 (1.42–1.51)	1.39 (1.37–1.42)	1.48 (1.44–1.52)	1.47 (1.45–1.50)	7.73	≤ 0.001
Blue Zone score	0	0.50	0	0		
4. Strong family ties and community support						
Married/partner: yes	0.70 (0.67–0.73)	0.78 (0.76–0.80)	0.72 (0.70–0.75)	0.70 (0.69–0.72)	15.13	≤ 0.001
Co–habits with someone: yes	0.68 (0.65–0.71)	0.77 (0.75–0.79)	0.71 (0.69–0.74)	0.71 (0.70–0.73)	10.53	≤ 0.001
Weekly contact with family members and/or relatives: yes	0.63 (0.59–0.66)	0.65 (0.63–0.67)	0.47 (0.44–0.50)	0.48 (0.46–0.50)	61.59	≤ 0.001
Weekly contact with friends and/or neighbours: yes	0.40 (0.37–0.43)	0.36 (0.34–0.38)	0.31 (0.28–0.33)	0.30 (0.29–0.32)	11.13	≤ 0.001
Has at least one confidant: yes	0.97 (0.96–0.98)	0.98 (0.97–0.99)	0.98 (0.97–0.99)	0.98 (0.98–0.99)	1.26	0.29
Volunteers: yes	0.34 (0.30–0.37)	0.47 (0.45–0.49)	0.29 (0.27–0.32)	0.36 (0.34–0.38)	41.23	≤ 0.001
Loneliness: yes	0.12 (0.10–0.14)	0.09 (0.07–0.10)	0.12 (0.10–0.14)	0.14 (0.13–0.15)	10.90	≤ 0.001
People in the neighbourhood have a positive attitude towards older people: yes	0.29 (0.26–0.33)	0.26 (0.24–0.28)	0.32 (0.29–0.35)	0.38 (0.36–0.40)	26.07	≤ 0.001
People in the neighbourhood have a positive attitude towards older people: yes	0.29 (0.26–0.33)	0.26 (0.24–0.28)	0.32 (0.29–0.35)	0.38 (0.36–0.40)	26.07	≤ 0.001
People in this area can be trusted: Strongly agree	0.47 (0.44–0.51)	0.42 (0.40–0.44)	0.39 (0.36–0.42)	0.47 (0.45–0.49)	10.30	≤ 0.001
Blue Zone score	0.10	0.50	0	0.30		
5. Respect the planet						
Agricultural activities: yes	0.54 (0.47–0.60)	0.58 (0.55–0.62)	0.54 (0.50–0.58)	0.56 (0.53–0.59)	0.99	0.40
Does gardening: yes	0.86 (0.82–0.90)	0.84 (0.81–0.86)	0.86 (0.83–0.89)	0.95 (0.93–0.97)	18.84	≤ 0.001
Blue Zone score	0	0	0	0.50		
6. A purpose in life						
Feels needed: yes	0.88 (0.86–0.90)	0.90 (0.89–0.92)	0.88 (0.86–0.90)	0.89 (0.88–0.90)	1.55	0.20
Meaningfulness (1–5)	4.00 (3.95–4.05)	4.06 (4.03–4.09)	3.99 (3.95–4.03)	3.99 (3.96–4.02)	4.56	≤ 0.001
Morale (0–17)	11.98 (11.74–12.22)	12.07 (11.93–12.22)	11.71 (11.52–11.90)	11.97 (11.84–12.09)	2.99	0.03
Attends religious activities: yes	0.23 (0.15–0.30)	0.41 (0.37–0.46)	0.38 (0.32–0.43)	0.46 (0.42–0.49)	10.66	≤ 0.001
Believes in a higher power: yes	0.60 (0.57–0.63)	0.78 (0.77–0.80)	0.78 (0.76–0.81)	0.88 (0.86–0.89)	105.43	≤ 0.001
Blue Zone score	0	0.40	0	0.40		
Environmental features						
Parks and green spaces close by: yes	0.80 (0.77–0.83)	0.77 (0.75–0.79)	0.74 (0.72–0.77)	0.64 (0.62–0.65)	47.99	≤ 0.001
Nature. Cabins. Water close by: yes	0.89 (0.86–0.92)	0.82 (0.80–0.84)	0.73 (0.70–0.76)	0.61 (0.59–0.63)	123.31	≤ 0.001
Beautiful landscape close by: yes	0.99 (0.96–1.01)	0.94 (0.93–0.96)	0.82 (0.80–0.84)	0.81 (0.80–0.83)	96.49	≤ 0.001
Own garden or shared green space: yes	0.88 (0.86–0.90)	0.89 (0.87–0.90)	0.87 (0.85–0.88)	0.90 (0.89–0.91)	3.67	0.01
Feels part of area: strongly agree	0.80 (0.77–0.83)	0.78 (0.76–0.80)	0.74 (0.72–0.77)	0.77 (0.76–0.79)	3.30	0.02
Blue Zone score	0.80	0	0	0.20		
Total Blue Zone score	**1.23**	**1.73**	**0**	**1.73**		
Health						
IADL dependency (0–4, higher score indicating more dependence)	0.80 (0.73–0.88)	0.81 (0.76–0.85)	0.64 (0.58–0.70)	0.73 (0.69–0.77)	7.34	≤ 0.001
PADL dependency: yes	0.07 (0.06–0.09)	0.06 (0.05–0.07)	0.037 (0.02–0.05)	0.035 (0.03–0.04)	10.00	≤ 0.001
I have visited the dentist during last year: yes	0.79 (0.76–0.82)	0.70 (0.68–0.72)	0.68 (0.66–0.71)	0.60 (0.58–0.62)	41.83	≤ 0.001
I have mostly my own teeth: yes	0.82 (0.79–0.85)	0.77 (0.75–0.79)	0.74 (0.72–0.77)	0.69 (0.67–0.70)	26.23	≤ 0.001
Self-rated health (1–5)	2.90 (2.84–2.97)	3.08 (3.03–3.12)	3.17 (3.12–3.23)	3.12 (3.09–3.16)	13.76	≤ 0.001
Pain during last week: yes	0.40 (0.36–0.43)	0.46 (0.44–0.49)	0.50 (0.47–0.53)	0.51 (0.49–0.53)	10.62	≤ 0.001
Medical conditions (0–5)	1.37 (1.30–1.44)	1.33 (1.29–1.37)	1.40 (1.35–1.46)	1.43 (1.39–1.46)	4.17	0.01
Number of medicines on a regular basis	3.23 (3.03–3.43)	3.29 (3.17–3.42)	3.60 (3.44–3.76)	4.06 (3.95–4.17)	32.73	≤ 0.001
I experience fatigue: yes	0.18 (0.15–0.21)	0.15 (0.13–0.17)	0.23 (0.21–0.26)	0.22 (0.20–0.23)	15.61	≤ 0.001
I have good memory: yes	0.49 (0.45–0.52)	0.41 (0.39–0.43)	0.39 (0.36–0.42)	0.38 (0.36–0.40)	83.72	≤ 0.001
Good health score	**5.0**	**2.0**	**2.0**	**1.0**		

*Note:* All results are adjusted for age, sex, educational level, and personal income. Each Blue Zone principle is awarded one point in total so that the contribution of each item is calculated by dividing 1 by the number of included items within each Blue Zone principle. A score is given when the region significantly differs from the other regions in terms of adherence to the Blue Zone lifestyle. By adding the scores, a total score for lifestyle principles and health indicators, respectively, was calculated and presented in bold text. The Blue Zone principle of ‘Ensure the legacy for future generations' was excluded since such variables are not included in the GERDA survey 2021/2022. Environmental features were included as an additional principle. Each Health indicator is awarded one point.

## Data Availability

The GERDA survey data from the wave 2021/2022 was stored at the Finnish Social Science Data Archive and can be accessed after an application process. More information is available here: https://urn.f/urn:nbn:f:fsd:T-FSD3819.

## Endnotes

^1^ Table 1 is based on descriptive official statistics on life expectancy and preliminary calculations of rates of centenarians and nonagenarians. These preliminary indicators of longevity do not reflect the rigorous demographic procedure involved in determining a validated longevous Blue Zone.

^2^More information about GERDA is available on the following website: https://www.gerdacenter.com/
